# Biodiesel Production by Acid Methanolysis of Slaughterhouse Sludge Cake

**DOI:** 10.3390/ani9121029

**Published:** 2019-11-25

**Authors:** Jung-Jeng Su, Yu-Chun Chou

**Affiliations:** 1 Dept. of Animal Science and Technology, National Taiwan University, Taipei 10673, Taiwan; jsu9155@gmail.com; 2 Bioenergy Research Center, College of Bio-resources and Agriculture, National Taiwan University, Taipei 10617, Taiwan

**Keywords:** slaughterhouse, sludge cake, acid methanolysis, biodiesel, fatty acid methyl ester

## Abstract

**Simple Summary:**

Excessive sludge in the wastewater treatment basins has to be removed periodically to ensure good water quality of the effluent. This study aims to evaluate the feasibility of biodiesel production by acid methanolysis of slaughterhouse sludge cake. Experimental and analytical results showed that acid methanolysis of sludge cake was one of the feasible and practical options to recycle sludge waste and produce renewable energy.

**Abstract:**

Biosludge is a normal form of accumulating microbial populations inside the sewage or wastewater treatment facilities. Excessive sludge in the wastewater treatment basins has to be removed periodically to ensure good water quality of the effluent. This study aims to evaluate the feasibility of biodiesel production by transesterification of slaughterhouse sludge cake. The sludge cake was collected from a selected commercial slaughterhouse and transesterified with methanol, *n*-hexane, and acids (e.g., sulfuric acid or hydrochloric acid) at 55 °C. Three acid concentrations (2%, 4%, and 8%, *v*/*v*) in methanol under four reaction time periods (4, 8, 16, and 24 h) were applied. Results showed that the highest accumulated fatty acid methyl ester (FAME) yields of 2.51 ± 0.08% and 2.27 ± 0.09% were achieved when 8% (*v*/*v*) of H_2_SO_4_ or HCl were added in a 4 h reaction time, respectively. Methyl esters of palmitic acid (C16:0), palmitoleic acid (C16:1), stearic acid (C18:0), and oleic acid (C18:1n9c) were the major components of biodiesel from acid methanolysis of slaughterhouse sludge cake. Experimental and analytical results of acid methanolysis of slaughterhouse sludge cake showed that acid methanolysis of sludge cake was one of the feasible and practical options to recycle sludge waste and produce renewable energy.

## 1. Introduction

In Taiwan, there were 4.6 million tons of agricultural biowastes (about 98% of the total amount of agricultural wastes) in 2017 [1]. Among these biowastes, the amount of livestock wastes was 2.28 million tons including animal manure, biosludge, and carcass (about 49% of the total amount of agricultural biowastes). Most agricultural biowastes were properly treated by means of landfill (30%), composting (52.5%), and other approaches (16.5%). Normally, the operating costs for sludge handling and disposal are in the range of 41–43% of the total operating costs of wastewater treatment [2]. If sludge stabilization is included, another 8–10% of the total operating costs have to be added. As a conclusion, we can state that sludge treatment is responsible for more than 50% of the total operating costs of wastewater treatment.

Biosludge has been used as the feedstock for producing biodiesel under various conditions with a solvent mixture solution (*n*-hexane:methanol:acetone = 3:1:1, *v*/*v*) or individual solvents [3]. Experimental results showed that the highest lipid extraction efficiency of the sludge was achieved by using the solvent mixture (27.43 ± 0.98%). The conversion efficiency of lipid to fatty acid methyl ester (FAME) was only 4.41 ± 0.63%. However, in situ transesterification of dried sludge resulted in a yield of 6.23%. The major cost for producing biodiesel (75–80% of total cost) from various resources was obtaining the feedstock [4,5]. 

Primary and secondary sludge samples obtained from a municipal wastewater treatment plant were freeze-dried and subjected to an acid-catalyzed in situ transesterification process [6]. Results indicated the maximum FAME yields obtained at 75 °C, 5% (*v*/*v*) H_2_SO_4_, and 12:1 methanol-to-sludge mass ratio were 14.5% and 2.5% for primary and secondary sludge, respectively. Gas chromatography (GC) analysis of the FAMEs revealed a similar fatty acid composition (e.g., C16:0 and C18:0 or C18:1) for both primary and secondary sludge. 

In situ transesterification of sludge with sulfuric acid as catalyst was studied by Revellame et al. [7]. After numerical optimization, they showed that an optimum biodiesel yield of 4.88% can be obtained at 55 °C, with a methanol-to-sludge ratio of 25:1, and sulfuric acid (4%, *v*/*v*). In the same study, an economic analysis showed that the in situ transesterification of wet activated sludge (84.5% moisture) was less economical than that of dried sludge (5% moisture).

The synthesized cationic polyacrylamide (PAM) was proven to improve sludge dewatering [8]. However, addition of PAM in the sludge cake might be a barrier for the transesterification of slaughterhouse sludge cake. In order to avoid the conventional preliminary step of sludge drying, dewatered municipal sludge (TS = 15%) was used as starting material. The best performance in FAME yield (18 wt%) with the lowest energy demand (17 MJ/kg FAME) was obtained by a new two-step approach with hexane extraction performed directly on dewatered acidified (H_2_SO_4_) sludge, followed by methanolysis of extracted lipids [9].

Lipids in the dried feedstock must be extracted prior to the transesterification process of fatty acids for conventional biodiesel production. Lipids extracted from four different types of wastewater sludge were applied to evaluate the suitability for biodiesel production. Results indicated that the primary sludge achieved the greatest lipids and biodiesel yields. The amount of extracted lipids for primary sludge was 25.3% compared to 21.9%, 10.1%, and 9.1% (dry wt) for blended, stabilized, and secondary sludge, respectively [10]. Moreover, the FAME yields were 13.9%, 10.9%, 2.9%, and 1% (dry wt) for primary, blended, secondary, and stabilized sludge, respectively. Gas chromatography analysis of the FAMEs revealed a similar fatty acids composition for all sludge tested with a predominance of palmitic acid (C16:0), stearic acid (C18:0), oleic acid (C18:1), and linoleic acid (C18:2). Comparison of sludge fatty acid profile with common biodiesel feedstocks (e.g., municipal wet sewage scum and brown grease from wastewater treatment plants) showed their suitability for the production of biodiesel [10,11,12]. Moreover, biofuel can be produced from hydrothermal carbonization of poultry slaughterhouse sludge cake under reaction temperatures of 170–220 °C [13].

Xylene can be an alternative cosolvent to hexane for transesterification to enhance the biodiesel yield from wet wastewater sludge. The water present in the sludge could be separated during transesterification by employing xylene. Xylene enhanced the biodiesel yield up to 8.12 ± 0.11% from wet wastewater sludge, which was 2.5 times higher than hexane (3.28 ± 0.04%) [14]. It was comparable to the maximum biodiesel yield of 9.68 ± 0.39% obtained from dried sludge with xylene as the cosolvent. The FAME content of the biodiesel increased approximately twofold by changing the cosolvent from hexane to xylene. However, xylene is carcinogenic to humans and it is better not to be used for transesterification of biosludge. Thus, the objective of this study was to test the feasibility of applying slaughterhouse sludge to produce biodiesel without lipid extraction and establish the optimal operation parameters for further applications.

## 2. Material and Methods

### 2.1. Feedstock for Acid Methanolysis

All sludge cakes were taken from the slaughterhouse of Yah-Sen Frozen Foods Co., Ltd., Taoyuan, Taiwan. The company owns the largest private slaughterhouse in northern Taiwan and its daily slaughter capacity is about two thousand pigs. The sludge cake was made from excessive sludge from the wastewater treatment facility of the slaughterhouse. For sludge cake production, PAM addition (1.1 mg/L PAM, w/v) enhanced the solid-like character of the sludge and promoted sludge dewatering efficiency by using a frame filter press. Sludge cake samples were taken and stored in a fridge at 4 °C for further analysis of total solids (TS) and volatile solids (VS). The frozen sludge cakes were placed in aluminum foil dishes and dried for 72 h at 65 °C to reduce water content. The dried sludge cake was then ground and the resulting powder was sieved by a 20 mm mesh screen. The dried sludge cake powder was then placed in zipper bags and preserved in dry areas.

### 2.2. Preliminary Test for Optimal Concentrations of Acid and Reaction Time Based on Accumulated FAME Yield

Various concentrations of acid catalyst were applied to evaluate FAME production of slaughterhouse sludge cake powder. Three different concentrations of H_2_SO_4_ (2%, 4%, and 8%, *v*/*v*) in methanol were used for sludge cake acid methanolysis. The 2%, 4%, and 8% (*v*/*v*) of H_2_SO_4_ in methanol was prepared by adding 10, 20, and 40 mL of H_2_SO_4_ into 500 mL methanol. Samples were taken at the 4th, 8th, 16th, and 24th h and analyzed for FAME yield individually.

### 2.3. Time Course Experiments for Accumulated FAME Yield from Acid Methanolysis of Sludge Cake Powder

Various concentrations of different acid catalysts were applied to evaluate FAME production of slaughterhouse sludge cake powder. Three different concentrations of H_2_SO_4_ or HCl (2%, 4%, and 8%, *v*/*v*) in methanol were used for acid methanolysis of sludge cake under 4, 8, 16, and 24 h periods.

It was found that sulfuric acid plays a key role in the process not only of acid methanolysis of glycerides but also in the production of new free fatty acids (FFAs) from soaps and their esterification with methanol [9]. Because of the limited solubility of FFAs in alcohol, a large amount of solvent is needed while applying the alcohol method extraction, which makes the process more complicated. The common pretreatment employed was esterification of the FFAs with methanol in the presence of sulfuric acid [15]. 

**Figure 1:**
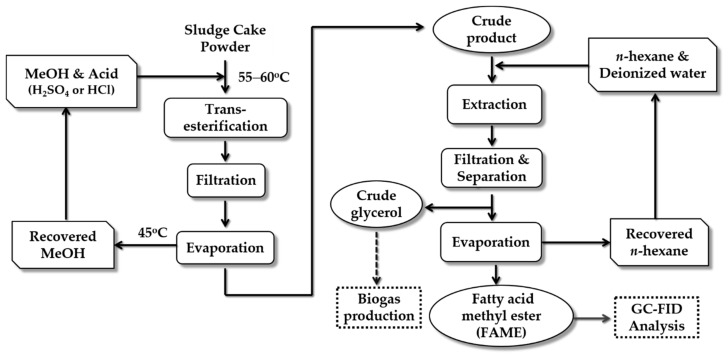
The flow chart of acid methanolysis process.

Thus, the acid methanolysis of sludge cake powder was modified from the conventional transesterification (Figure 1). There were five steps included in the acid methanolysis process as follows: (1) Acid methanolysis: Dried sludge cake powder (20 g) was placed in a 1 L screw-cap glass flask. Methanol (500 mL) was added into the screw-cap glass flasks of individual sets with different addition volumes of acids (e.g., concentrate H_2_SO_4_ or HCl) in triplicates. The 2%, 4%, and 8% (*v*/*v*) of either H_2_SO_4_ or HCl in methanol were prepared by adding 10, 20, and 40 mL of either H_2_SO_4_ or HCl into 500 mL methanol, respectively. The screw-cap flasks with acid sludge cake liquid were placed in a water bath at 55–60 °C with 200 rpm stirring and samples of about 120 mL were taken from the flasks at the time periods of 4, 8, 16, and 24 h, individually. (2) Filtration: After the liquid samples had been cooled to room temperature, these were centrifuged at 4000 rpm for 15 min. The supernatant was then paper (No. 61631, 1 μm × 47 mm, Pall Co., USA) filtered under vacuum and the filtrate was kept for the next step. (3) Evaporation: Rotary evaporator (BÜCHI R-200, Switzerland) was applied to recover methanol in a water bath (BÜCHI B-490, Switzerland) at 45 °C. (4) Extraction: The liquid after the methanol recovery process was then placed in 250 mL screw-cap flasks with 75 mL n-hexane added for FAME extraction. Deionized water (25 mL) was used to wash the extract liquid by stirring at 600 rpm for 40 min. (5) Filtration: The extracted liquid was left to rest for 5 min for layer separation containing upper layer (FAME layer) and lower layer (crude glycerol layer). The upper layer (45 mL) was collected and placed into 50 mL centrifuge tubes for centrifugation (4,000 rpm, 6 min). After centrifugation, the upper layer (20 mL) liquid was paper filtered (Advantec No.7) with 10 g of dehydrate Na_2_SO_4_ powder for removing water from the liquid samples. (6) Evaporation: Rotary evaporator (BÜCHI R-200, Switzerland) was applied to recover n-hexane in a water bath (BÜCHI B-490, Switzerland) at 55 °C. The liquid samples were analyzed for concentrations of FAME by gas chromatography with flame ionization detector (GC/FID).

### 2.4. Qualitative and Quantitative Analysis of Fatty Acid Methyl Ester (FAME)

The acid methanolysis samples were analyzed for their composition and yield of FAME by using gas chromatography (Agilent Technologies GC 7820A, USA), which was equipped with a flame ionization detector (FID) and SP-2380 capillary column (60 m × 0.25 mm × 0.5 µm film thickness; Supelco Analytical of Sigma-Aldrich Co., PA, USA). Nitrogen gas was used as carrier gas with a flow rate of 12 mL/min. The oven temperature was 70 °C (2 min) → 190 °C (3 min) with 4 °C/min → 210 °C (2 min) with 3 °C/min → 240 °C (2 min) with 3 °C/min → 270 °C (2 min) with 5 °C/min. The injector and detector temperatures were set at 250 and 260 °C, respectively. Sample injection volume was 1 µL and split ratio was 1:30. Calibration curves of the FAMEs were obtained by external standard method using the standard 37 Component FAME Mix (CRM47885, Supelco, USA) (the calibration curves’ correlation coefficients were >0.995). Based on the calibration curve of the FAME standard, the contents of the acid methanolysis samples were obtained in the unit of mg/mL. The total FAME yield (C12 to C22 of FAME) from the acid methanolysis samples was calculated by the following equation:

$$Total\;FAME\;yield\;(\%,\;w/w)=\frac{F\times H}{S}\times \frac{1\;mg}{1000\;µg}\times 100\%$$

F: Total amount of the FAME from the acid methanolysis samples (µg/mL)
H: Amount of *n*-hexane used for each batch (75 mL)
S: Amount of dried sludge cake powder for each sampling (5000 mg)

### 2.5. Statistical Analysis

The analysis of variance (ANOVA) was performed using Prism 6 software to compare the experimental results using one-way ANOVA and Tukey’s test for multiple comparison among various factors.

## 3. Results and Discussion

### 3.1. Characterization of Slaughterhouse Sludge Cake

Analytical results showed that the moisture, total solids (TS), and volatile solids (VS) of the original slaughterhouse sludge cake was 73.2 ± 1.4%, 27.0 ± 1.4%, and 81.8 ± 0.3%, respectively. The values of VS are on a dried basis.

### 3.2. Preliminary Test for Optimal Concentrations of H_2_SO_4_ and Reaction Time Based on Accumulated FAME Yield

**Figure 2:**
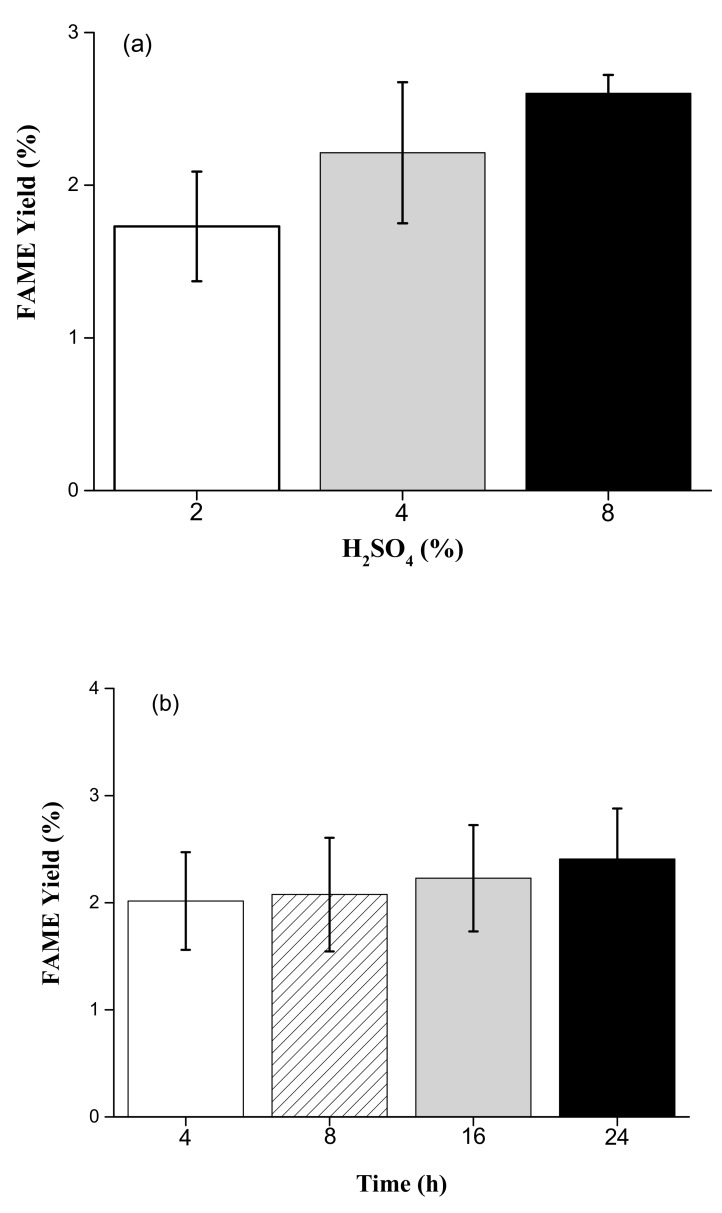
(**a**) Effects of different H_2_SO_4_ concentrations on FAME yield in a 24 h period (*p* < 0.05). (**b**) Comparison of different reaction time periods on FAME yield with 4% H_2_SO_4_ (*p* > 0.05).

The FAME yields of 2%, 4%, and 8% H_2_SO_4_ were 1.73 ± 0.36%, 2.21 ± 0.46%, and 2.60 ± 0.12%, in a 24 h reaction time, respectively. For the interaction effect of acid concentrations in a 24 h reaction time, results showed that there was significant difference among the FAME yields of the three experimental sets (2%, 4%, and 8% of H_2_SO_4_) (*p* < 0.05) (Figure 2a). However, the FAME yields from using 4% H_2_SO_4_ were 2.02 ± 0.46%, 2.08 ± 0.53%, 2.23 ± 0.50%, and 2.41 ± 0.47% in 4, 8, 16, and 24 h reaction times, respectively. For the time course experiments under different reaction time periods (4, 8, 16, and 24 h) with 4% H_2_SO_4_, however, results showed that there was no significant difference among the FAME yields of the three experimental sets (*p* > 0.05) (Figure 2b). Results showed that the highest FAME yield by acid methanolysis of sludge cake powder was with 8% H_2_SO_4_.

### 3.3. Acid Methanolysis of Sludge Cake with Different Concentrations of Sulfuric Acid and Hydrochloric Acid 

**Figure 3:**
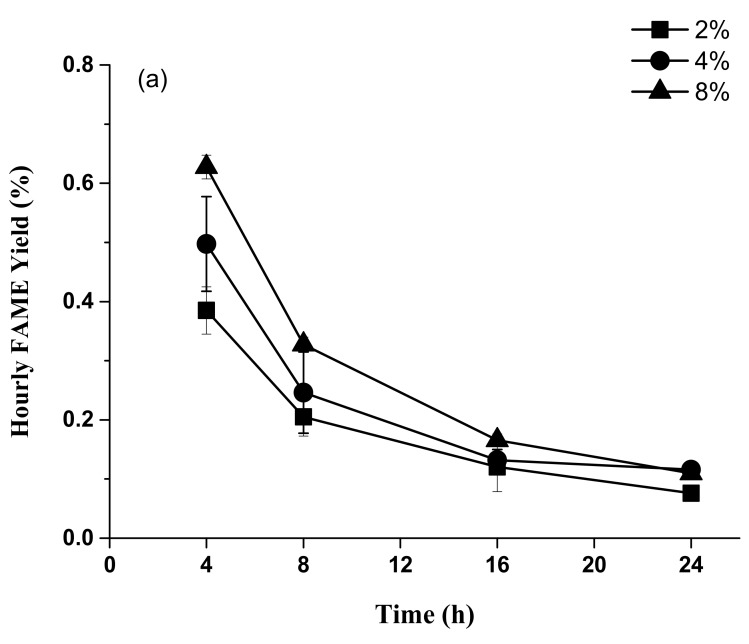

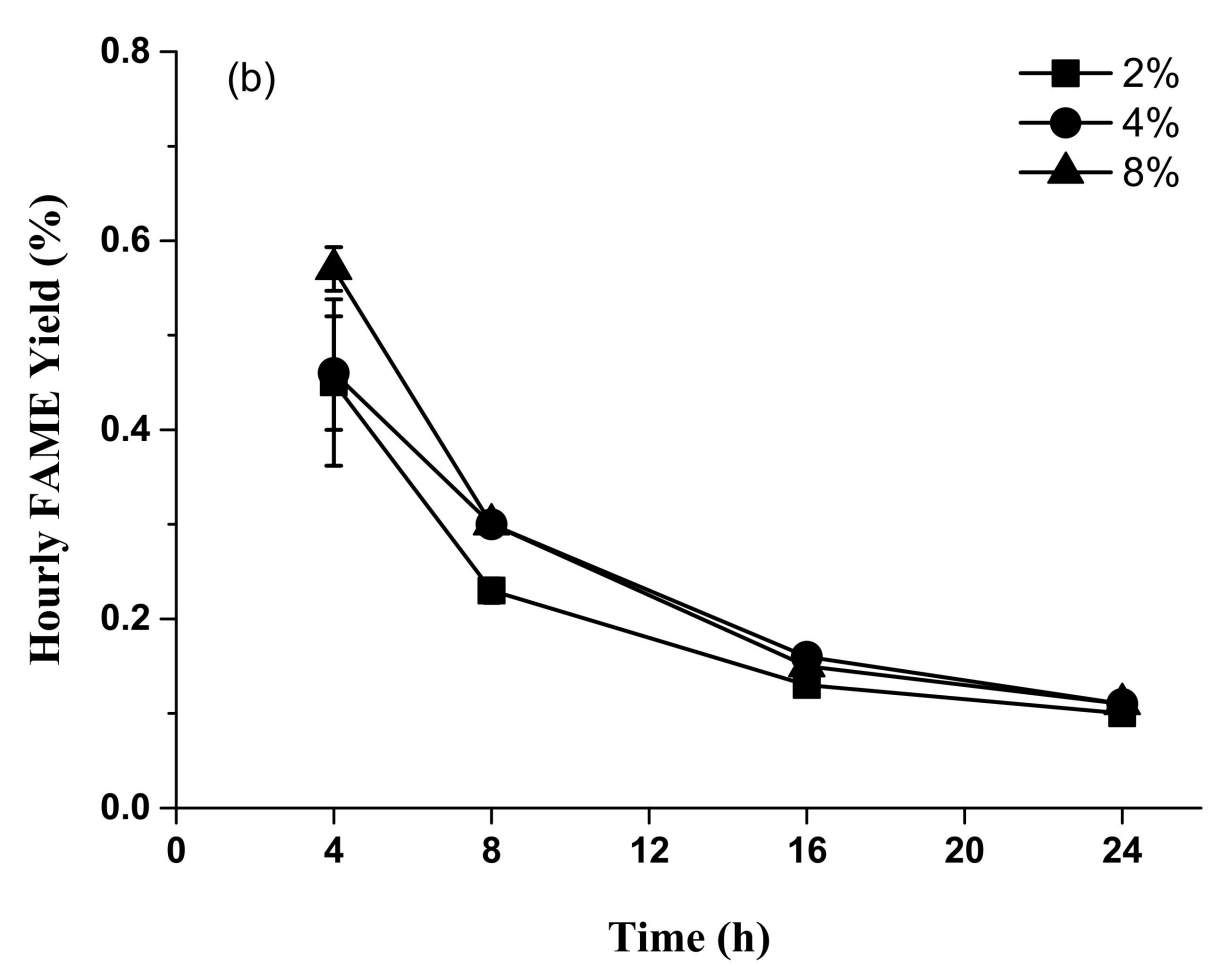
Effects of different H_2_SO_4_ (**a**) and HCl (**b**) concentrations on hourly FAME yield.

When using H_2_SO_4_, the hourly FAME yields of the 8% set were 0.63 ± 0.02%, 0.33 ± 0.01%, 0.17 ± 0.01%, and 0.11 ± 0.01% in the 4, 8, 16, and 24 h time periods, respectively. Additionally, the hourly FAME yields of the 4% and 2% H_2_SO_4_ sets were 0.50 ± 0.08%–0.12 ± 0.01% and 0.39 ± 0.01%–0.08 ± 0.01% in the 4, 8, 16, and 24 h time periods, respectively. The 4 h reaction time achieved a higher hourly FAME yield than the other reaction time periods (Figure 3a). 

Similarly, when using HCl, the hourly FAME yields of the 8% set were 0.57 ± 0.02%, 0.30 ± 0.01%, 0.15 ± 0.00%, and 0.11 ± 0.00% in the 4, 8, 16, and 24 h time periods, respectively. The hourly FAME yield of the 4% and 2% HCl sets were 0.46 ± 0.06%–0.11 ± 0.00% and 0.45 ± 0.09%–0.10 ± 0.01% in the 4, 8, 16, and 24 h time periods, respectively. The 4 h reaction time also achieved a higher hourly FAME yield than the other reaction time periods (Figure 3b).

**Figure 4:**
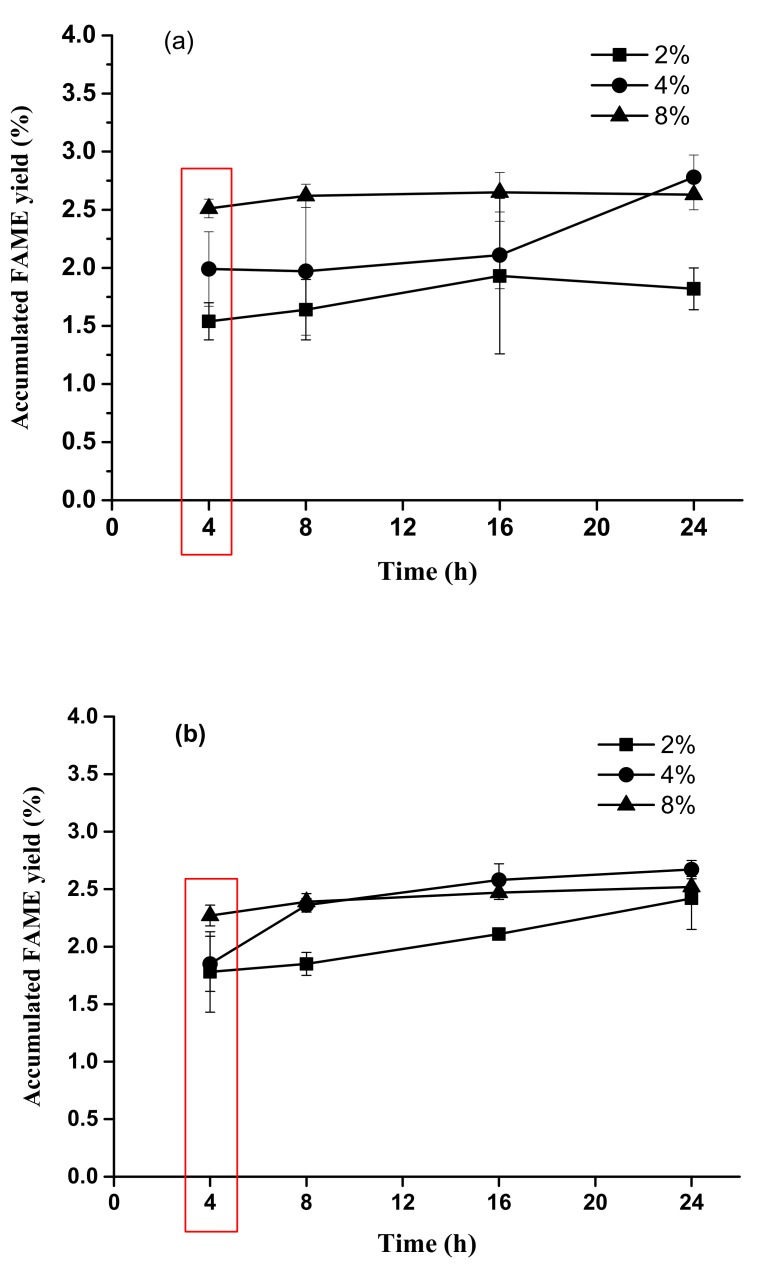
Effects of different H_2_SO_4_ (**a**) and HCl (**b**) concentrations on accumulated FAME yield.

For estimating the accumulated FAME yield by using 8% H_2_SO_4_, the FAME yields were 2.51 ± 0.08%, 2.62 ± 0.10%, 2.65 ± 0.17%, and 2.63 ± 0.13% in the 4, 8, 16, and 24 h time periods, respectively (*p* > 0.05). The accumulated FAME yields of the 4% and 2% H_2_SO_4_ sets were 1.99 ± 0.32%–2.78 ± 0.19% and 1.54 ± 0.06%–1.93 ± 0.67% in the 4, 8, 16, and 24 h time periods, respectively. The 4 h reaction time was the optimal time period to achieve a higher accumulated FAME yield than the other reaction time periods (Figure 4a).

When using 8% HCl, the accumulated FAME yields were 2.27 ± 0.09%, 2.39 ± 0.07%, 2.47 ± 0.06%, and 2.52 ± 0.09% in the 4, 8, 16, and 24 h time periods, respectively (*p* > 0.05). The accumulated FAME yields of the 4% and 2% HCl sets were 1.85 ± 0.24%–2.67 ± 0.08% and 1.78 ± 0.35%–2.42 ± 0.27% in the 4, 8, 16, and 24 h time periods, respectively. The 4 h reaction time was the optimal time period to achieve a higher accumulated FAME yield than the other reaction time periods (Figure 4b). Experimental results showed that there was no significant difference among the various reaction time periods (4, 8, 16, and 24 h periods) (*p* > 0.05) with 8% H_2_SO_4_ or HCl for accumulated FAME yield (Figure 4). For stable acid methanolysis of slaughterhouse sludge cake, a stable thermostat and agitation of the sludge cake were required during the whole process. Results showed that the highest rates of FAME yield were achieved in a 4 h time period for all experimental sets (2%, 4%, and 8% sulfuric acid) (Figure 3). FAME yield efficiency rapidly declined with increased reaction time after 4 h. Experimental results imply that the optimal reaction time for the acid methanolysis of slaughterhouse sludge cake using sulfuric acid is 4 h. 

There are four primary factors affecting the yield of biodiesel: alcohol quantity, reaction time, reaction temperature, and acid concentration. The free fatty acids (FFAs) can react with alcohol to form ester (biodiesel) by a transesterification reaction. Normally, the catalyst for this reaction is concentrated sulfuric acid. Due to the slow reaction rate and the high methanol-to-oil molar ratio required, acid-catalyzed acid methanolysis has not gained as much attention as alkali-catalyzed acid methanolysis [15]. From the results of the 8% H_2_SO_4_ or HCl set, most acid methanolysis processes might be completed in 4 h because of slow increased FAME yield after 4 h. Moreover, the accumulated FAME yields for all experimental sets were not significantly different (*p* > 0.05), which implies that lipids were completely transesterified and the reaction achieved a steady state (Figure 4).

For hourly FAME yield, the highest hourly FAME yield was achieved in 4 h and significantly decreased after 4 h for all sets by either 8% H_2_SO_4_ or HCl (*p* < 0.05). Most lipid acid methanolysis might be completed after 16 h for all sets (Figure 3). For each sampling point, however, there was no significant difference in FAME production rates among the different concentrations of H_2_SO_4_ or HCl (*p* > 0.05). The pH, moisture, TS, and VS (in dry basis) of the crude glycerol was 6.83, 75.56%, 24.44%, and 54.42%, respectively [16]. Moreover, the crude glycerol of this study was recycled and identified for biogas production with dairy wastewater [16]. 

### 3.4. Comparison of FAME Components from Acid Methanolysis of Sludge Cake with Different Concentrations of H_2_SO_4_ and HCl

**Figure 5:**
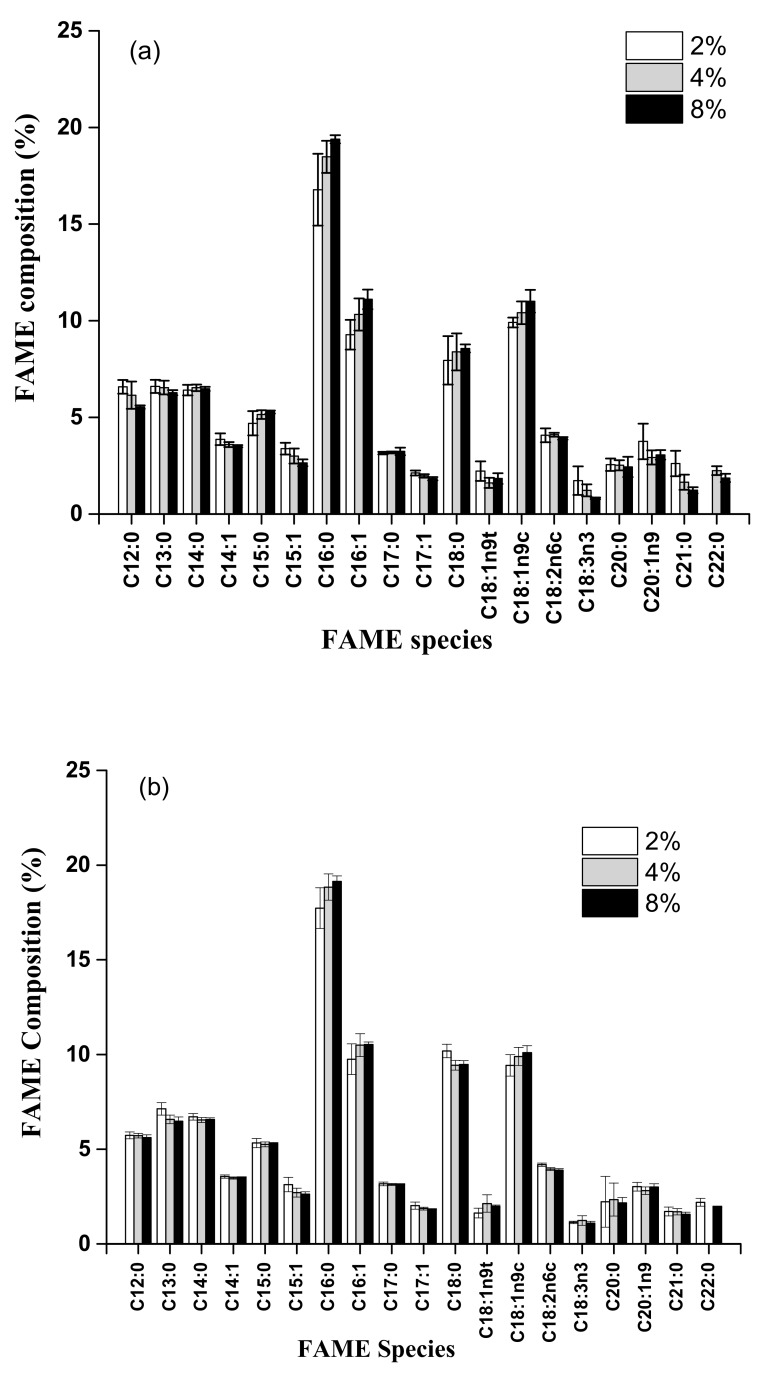
FAME composition of the methanolized slaughterhouse sludge cake with different H_2_SO_4_ (**a**) and HCl (**b**) concentrations.

Analytical results of FAME samples with 8% H_2_SO_4_ showed that the concentrations of palmitic acid (C16:0), palmitoleic acid (C16:1), stearic acid (C18:0), and oleic acid (C18:1n9c) were 19.4 ± 0.21%, 11.1 ± 0.50%, 8.57 ± 0.21%, and 11.0 ± 0.59%, respectively. The 4% H_2_SO_4_ set showed that the concentrations of palmitic acid, palmitoleic acid, stearic acid, and oleic acid were 18.5 ± 0.83%, 10.3 ± 0.83%, 8.40 ± 0.96%, and 10.4 ± 0.59%, respectively. Moreover, the 2% H_2_SO_4_ set showed that the concentrations of palmitic acid, palmitoleic acid, stearic acid, and oleic acid were 16.8 ± 1.86%, 9.28 ± 0.77%, 7.95 ± 1.25%, and 9.91 ± 0.26%, respectively (Figure 5a).

Similarly, analytical results of FAME samples with 8% HCl showed that the concentrations of palmitic acid, palmitoleic acid, stearic acid, and oleic acid were 19.1 ± 0.29%, 10.5 ± 0.14%, 9.47 ± 0.20%, and 10.1 ± 0.36%, respectively. The 4% HCl set showed that the concentrations of palmitic acid, palmitoleic acid, stearic acid, and oleic acid were 18.8 ± 0.69%, 10.5 ± 0.61%, 9.43 ± 0.25%, and 9.90 ± 0.48%, respectively. Moreover, the 2% HCl set showed that the concentrations of palmitic acid, palmitoleic acid, stearic acid, and oleic acid were 17.7 ± 1.07%, 9.75 ± 0.81%, 10.2 ± 0.35%, and 9.42 ± 0.56%, respectively (Figure 5b).

Thus, the major FAME components from the acid methanolysis of sludge cake were palmitic acid, palmitoleic acid, stearic acid, and oleic acid independently of which concentrations of H_2_SO_4_ or HCl were applied for all experimental sets (Figure 5). Moreover, major forms of the FAMEs were palmitic acid, oleic acid, palmitoleic acid, and stearic acid based on the concentrations in sequence for all experimental sets. Thus, concentrations of acid catalyst did not affect the composition of the FAMEs, but the fatty acids profile of the sludge cake could affect the components of FAMEs.

Finally, analytical results showed that major FAME components were palmitic acid (C16:0), palmitoleic acid (C16:1), stearic acid (C18:0), and oleic acid (C18:1) with either H_2_SO_4_ or HCl as the acidic catalyst under the same conditions (Figure 5). Thus, components of the FAMEs were not affected by the type of catalyst, concentrations of acid catalyst, or reaction time of acid methanolysis.

Results showed that the accumulated FAME yield was 2.51% or 2.27% with 8% of H_2_SO_4_ or HCl, respectively, in a 4 h reaction time period (Table 1). It was lower than the accumulated FAME yield using municipal primary sewage sludge (14.5%) with 5% H_2_SO_4_ in an 8 h reaction time period [6]. However, the accumulated FAME yield of this study was higher than other studies using secondary sewage sludge (3.95–6.23%) with 1–10% H_2_SO_4_ in a 24 h reaction time period [3,7,17]. Thus, the types of sludge and concentrations of H_2_SO_4_ may affect the accumulated FAME yield.

Primary sludge was normally less degraded by microorganisms and contained higher lipid content. Thus, more FAMEs can be produced from primary sludge by the transesterification process. Secondary-sewage sludge was normally the excessive activated sludge from aeration basins which contains biomass of the microorganisms instead of organics including lipids. Hence, less FAMEs can be produced from secondary sludge by the transesterification process. The slaughterhouse sludge cake was mainly composed of waste anaerobic and activated sludge with less lipid content than primary sludge. However, results of this study showed that the accumulated FAME yield was still higher than that with secondary sewage sludge (Table 1).

Results of this study showed that the main components of FAME, palmitic acid, palmitoleic acid, stearic acid, and oleic acid, were the same as in other studies [10,11,17]. This means that the free fatty acid profile of different sludge sources was almost identical to slaughterhouse sludge cake. The FAME contents from the slaughterhouse sludge were similar to those from pork oil with a higher content of saturated and monounsaturated fatty acids. Thus, the FAME from slaughterhouse sludge was relatively stable with regards to oxidation, but with higher cloud point and pour point characterization. This characterization could affect the cold flow properties of FAME and be compensated by mixing conventional diesel, antifreeze, or branched alcohols [10]. Recently, wet sewage scum has been an innovative source for biodiesel production under mild conditions (72 °C) [11]. Also, a brown grease from municipal wastewater treatment plants with about 50% of free fatty acids (FFAs) may be efficiently converted into FAMEs under mild conditions (47 °C and atmospheric pressure) [12].

### 3.5. Methanol Recovery from Acid Methanolysis of Sludge Cake with Different Concentrations of Sulfuric Acid and Hydrochloric Acid 

**Figure 6:**
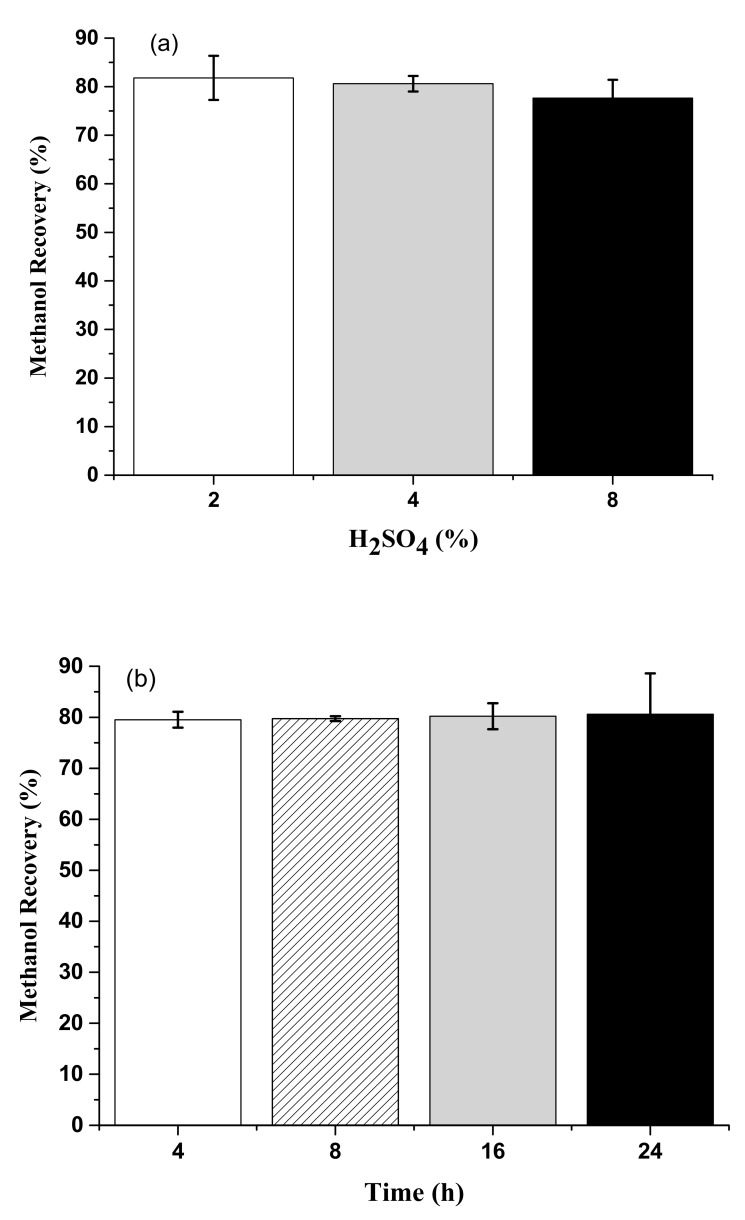
(**a**) Effects of different H_2_SO_4_ concentrations on methanol recovery efficiency (*p* > 0.05). (**b**) Effects of different reaction time periods on methanol recovery efficiency with H_2_SO_4_ as the catalyst (*p* > 0.05).

**Figure 7:**
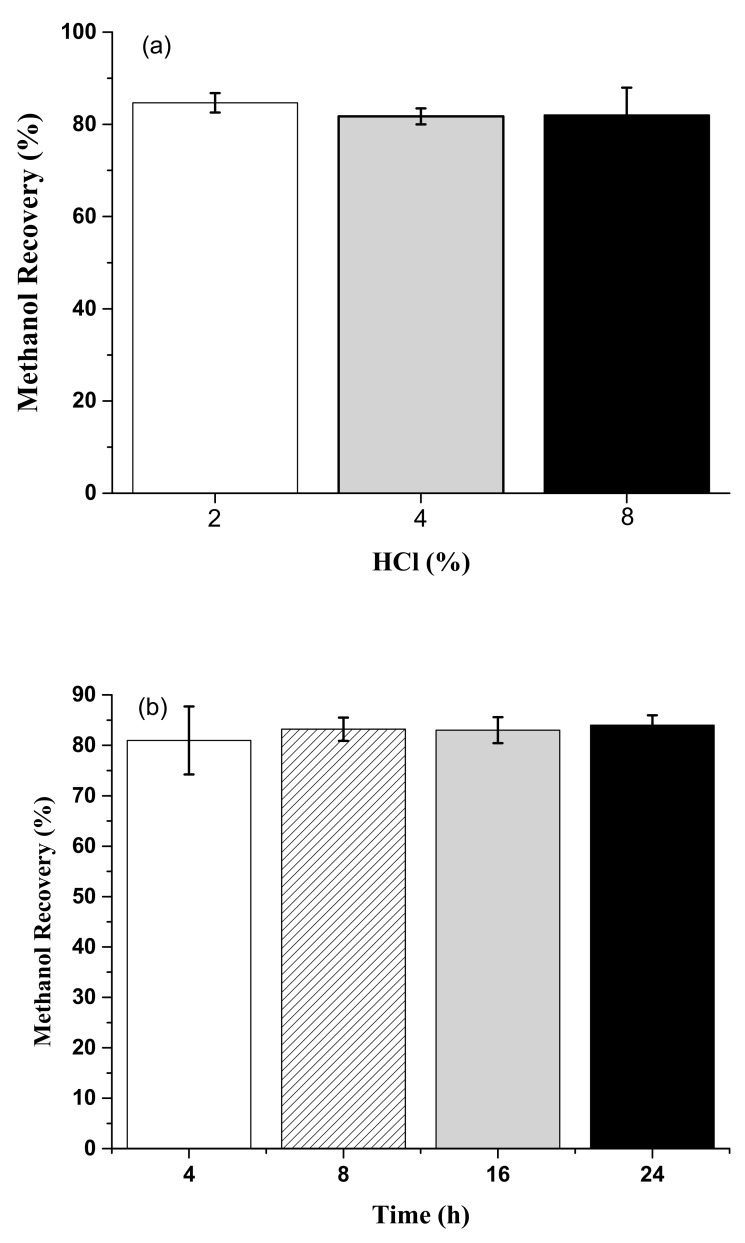
(**a**) Effects of different HCl concentrations on methanol recovery efficiency (*p* > 0.05). (**b**) Effects of different reaction time periods on methanol recovery efficiency with HCl as the catalyst (*p* > 0.05).

The average methanol recovery efficiencies of the 2%, 4%, and 8% H_2_SO_4_ sets were 81.8 ± 4.54%, 80.6 ± 1.60%, and 77.65 ± 3.75%, respectively (Figure 6a). The average methanol recovery efficiencies among the H_2_SO_4_ sets were 79.5 ± 1.55%, 79.7 ± 0.46%, 80.2 ± 2.55%, and 80.6 ± 8.00%, respectively, in the 4, 8, 16, and 24 h reaction times (*p* > 0.05) (Figure 6b). Moreover, the average methanol recovery efficiencies of the 2%, 4%, and 8% HCl sets were 84.7 ± 2.10%, 81.7 ± 1.73%, and 82.0 ± 5.95%, respectively (Figure 7a). The average methanol recovery efficiencies among the HCl sets were 81.0 ± 6.74%, 83.2 ± 2.3%, 83.0 ± 2.59%, and 84.0 ± 1.96%, respectively, in the 4, 8, 16, and 24 h reaction times (*p* > 0.05) (Figure 7b). 

**Figure 8:**
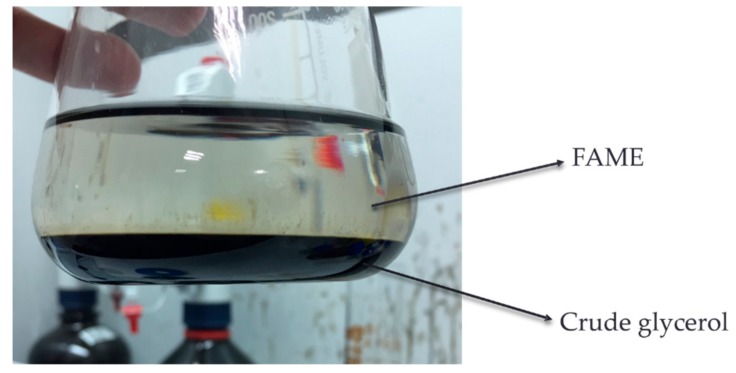
Appearance of the separated layers after FAME extraction by *n*-hexane.

Results showed that methanol recovery efficiency was more than 80%, independently of the type of acids (e.g., H_2_SO_4_ and HCl), concentrations of acid (2%, 4%, and 8% of H_2_SO_4_ or HCl), or reaction time (4, 8, 16, and 24 h periods) of sludge cake acid methanolysis. This achievement can assist cost reduction of methanol for industrialization. Consumption of methanol might result from the acid methanolysis process and methanol emission in the air. Besides, results showed that the highest hourly and accumulated FAME yields were achieved with 8% acids (either H_2_SO_4_ or HCl) in a 4 h reaction time. Results demonstrated that there was adequate reaction time for acid methanolysis of most lipids to FAME in 4h. The appearance of FAME and crude glycerol is shown in Figure 8.

## 4. Conclusions

In this study, biodiesel (i.e., FAME) was produced by the acid methanolysis of sludge cake with methanol and *n*-hexane. The optimal concentration of acids, H_2_SO_4_ or HCl, and reaction time were 8% and 4 h, respectively, to achieve the highest hourly and accumulated FAME yield. Results showed that the main components of FAME, palmitic acid (C16:0), palmitoleic acid (C16:1), stearic acid (C18:0), and oleic acid (C18:1n9c), were the same as in other similar studies. This indicates that the free fatty acid profile of different sludge sources was almost the same as in slaughterhouse sludge cake. Because the sulfur residue in the crude FAME may result in emission of SO_2_ after combustion, HCl is preferred to be the optimal acid catalyst. The FAME can be the fuel for conventional burners. Moreover, the crude glycerol has been recycled to produce biogas by anaerobic digestion of crude glycerol and has proven that slaughterhouse sludge cake is a feasible feedstock for producing biogas through anaerobic codigestion. Cost-effective biodiesel recovery from slaughterhouse wastewater sludge mixed with the lipid-rich primary sludge after slaughtering might become a method of converting negative value waste into a high-value product in the near future.

## Figures and Tables

**Table 1:** Comparison of the results of producing biodiesel from different types of sludge by various methods.

Sources of Sludge	Catalysts & Concentrations	Addition of MeOH (mL/g)	Reaction Temp. (°C)	Reaction Time (h)	Accumulated FAME Yield (%, *w*/*w*)	References
Municipal wastewater treatment plant (secondary sludge)	H_2_SO_4_ (1%)	5	50	24	6.23	Dufreche et al. [3]
Municipal wastewater treatment plant (primary sludge)	H_2_SO_4_ (5%)	12	75	8	14.5	Mondala et al. [6]
Municipal wastewater treatment plant (secondary sludge)	H_2_SO_4_ (5%)	12	75	8	2.5	
Municipal wastewater treatment plant (secondary sludge)	H_2_SO_4_ (4%)	30	55	24	4.79	Revellame et al. [7]
Municipal wastewater treatment plant (secondary sludge)	H_2_SO_4_ (10%)	30	75	24	3.93	Revellame et al. [17]
Slaughterhouse sludge cake	H_2_SO_4_ (8%)	25	55	4	2.51	This study (Su and Chou)
	HCl (8%)	25	55	4	2.27	

## References

[1] DGBAS (2017). Green National Income. https://ebook.dgbas.gov.tw/public/Data/9191653488VPAUQXD.pdf.

[2] Kroiss H. (2004). What is the potential for utilizing the resources in sludge?. Water Sci. Technol..

[3] Dufreche S., Hernandez R., French T., Sparks D., Zappi M., Alley E. (2007). Extraction of lipids from municipal wastewater plant microorganisms for production of biodiesel. J. Am. Oil Chem. Soc..

[4] You Y.D., Shie J.L., Chang C.Y., Huang S.H., Pai C.Y., Yu Y.H., Chang C. (2008). Economic cost analysis of biodiesel production: Case in soybean oil. Energ. Fuels.

[5] Knothe G., Razon L. (2017). Biodiesel fuels. Prog. Energy Combust. Sci..

[6] Mondala A., Liang K., Toghiani H., Hernandez R., French T. (2009). Biodiesel production by in situ transesterification of municipal primary and secondary sludges. Bioresour. Technol..

[7] Revellame E., Hernandez R., French W., Holmes W., Alley E. (2010). Biodiesel from activated sludge through in situ transesterification. J. Chem. Technol. Biotechnol..

[8] Wang F.H., Wang H.J., Hu H., Zeng R.J. (2017). Applying rheological analysis to understand the mechanism of polyacrylamide (PAM) conditioning for sewage sludge dewatering. RSC Adv..

[9] Pastore C., Lopez A., Lotito V., Mascolo G. (2013). Biodiesel from dewatered wastewater sludge: A two-step process for a more advantageous production. Chemosphere.

[10] Olkiewicz O., Fortuny A., Stüber F., Fabregat A., Font J., Bengoa C. (2012). Evaluation of different sludges from WWTP as a potential source for biodiesel production. Procedia Eng..

[11] di Bitonto L., Lopez A., Mascolo G., Mininni G., Pastore C. (2016). Efficient solvent-less separation of lipids from municipal wet sewage scum and their sustainable conversion into biodiesel. Renew. Energ..

[12] Pastore C., Lopez A., Mascolo G. (2014). Efficient conversion of brown grease produced by municipal wastewater treatment plant into biofuel using aluminum chlorine hexahydrate under very mild conditions. Bioresour. Technol..

[13] Oh S.-Y., Yoon Y.-M. (2017). Energy recovery efficiency of poultry slaughterhouse sludge cake by hydrothermal carbonization. Energies.

[14] Choi O.K., Song J.S., Cha D.K., Lee J.W. (2014). Biodiesel production from wet municipal sludge: Evaluation of in situ transesterification using xylene as a cosolvent. Bioresour. Technol..

[15] Leung D., Wu X., Leung M.K.H. (2010). A review on biodiesel production using catalyzed transesterification. Appl. Energy..

[16] Chou Y.C., Su J.J. (2019). Biogas production by anaerobic co-digestion of dairy wastewater with the crude glycerol from slaughterhouse sludge cake transesterification. Animals.

[17] Revellame E., Hernandez R., French W., Holmes W., Alley E., Robert C. (2011). Production of biodiesel from wet activated sludge. J. Chem. Technol. Biotechnol..

